# Characterization of the complete chloroplast genome of *Rumex nepalensis* (Polygonaceae)

**DOI:** 10.1080/23802359.2020.1778568

**Published:** 2020-06-16

**Authors:** Chun-Xia Wu, Cai-Cai Zhai, Shou-Jin Fan

**Affiliations:** Key Lab of Plant Stress Research, College of Life Sciences, Shandong Normal University, Ji’nan, China

**Keywords:** Plastome, phylogeny, *Rumex nepalensis*

## Abstract

*Rumex nepalensis* (Polygonaceae) is a fairly common perennial herb of high altitudes. In this study, we determined the complete chloroplast genome (plastome) of *R. nepalensis* with genome-skimming method. The complete plastome of *R. nepalensis* was 159,110 bp in length with a quadripartite structure, including a large single-copy region of 84,810 bp, a small single-copy region of 13,044 bp, and a pair of inverted repeats regions of 30,628 bp. The overall guanine-cytosine (GC) content was 37.5%. A total of 112 unique genes was annotated in this plastome, including 78 protein-coding genes, 30 tRNA genes, and four rRNA genes. In the ML tree, *R. nepalensis* was sister to *R. crispus*, and *Rumex* was sister to a clade comprising *Rheum* and *Oxyria* within Polygonaceae famliy.

*Rumex nepalensis* (Polygonaceae) is a fairly common perennial herb of high altitudes (Farooq et al. 2013). It grows between 800–4000 m in Afghanistan, China, India, Indonesia, Japan, Myanmar, Nigeria, Nepal, Pakistan, Tajikistan, Vietnam, South-west Asia, Turkey, Bhutan and South Africa (Kumar et al. 2018). For thousands of years, *R. nepalensis* has been served as the basis of traditional medicine systems in Nigeria, India, China and Indonesia (Ahmad et al. 2012; Yi et al. 2012; Kumar et al. 2018). It can also be used as vegetable, forage, coloring agent, and flavoring (Venkataramegowda 2012; Kumar et al. 2018). In this study, we reported the plastome of *R. nepalensis* for resolving its phylogenetic position.

Fresh leaves of *R. nepalensis* were sampled from Kunming Botanic Garden, Chinese Academy of Sciences (Yunnan, China; 25°8′N, 102°44′E). Voucher specimen (SD251) was deposited at College of Life Sciences, Shandong Normal University. Total genomic DNA was extracted using the modified CTAB method as described in Qu, Fan, et al. (2019). Total genomic DNA was then sequenced with the Novaseq platform. Plastome assembly was conducted with Organelle Genome Assembler (OGA) (Qu, Fan, et al. 2019). Plastome annotation was conducted with Plastid Genome Annotator (PGA) (Qu, Moore, et al. 2019), by manual correction with Geneious v9.1.4. The complete plastome was submitted to GenBank with accession number MT457825.

The complete plastome of *R. nepalensis* was 159,110 bp in length with a quadripartite structure, including a large single-copy region of 84,810 bp, a small single-copy region of 13,044 bp, and a pair of inverted repeats regions of 30,628 bp. The overall guanine-cytosine (GC) content was 37.5%. A total of 112 unique genes was annotated in this plastome, including 78 protein-coding genes, 30 tRNA genes, and four rRNA genes. Among them, ten protein-coding genes (atpF, ndhA, ndhB, petB, petD, rpl2, rpl16, rpoC1, rps12, and rps16) and six tRNA genes (trnA-UGC, trnG-UCC, trnI-GAU, trnK-UUU, trnL-UAA, and trnV-UAC) contained one intron, and two protein-coding genes (clpP and ycf3) contained two introns.

To determine the phylogenetic position of *R. nepalensis*, a maximum likelihood tree of 14 Polygonaceae species (Figure 1) was reconstructed with RAxML v8.2.10 (Stamatakis 2014), based on alignment of all shared genes with MAFFT v7.313 (Katoh and Standley 2013). In the ML tree, *R. nepalensis* was sister to *R. crispus*, and *Rumex* was sister to a clade comprising *Rheum* and *Oxyria* within Polygonaceae famliy.

**Figure 1.**
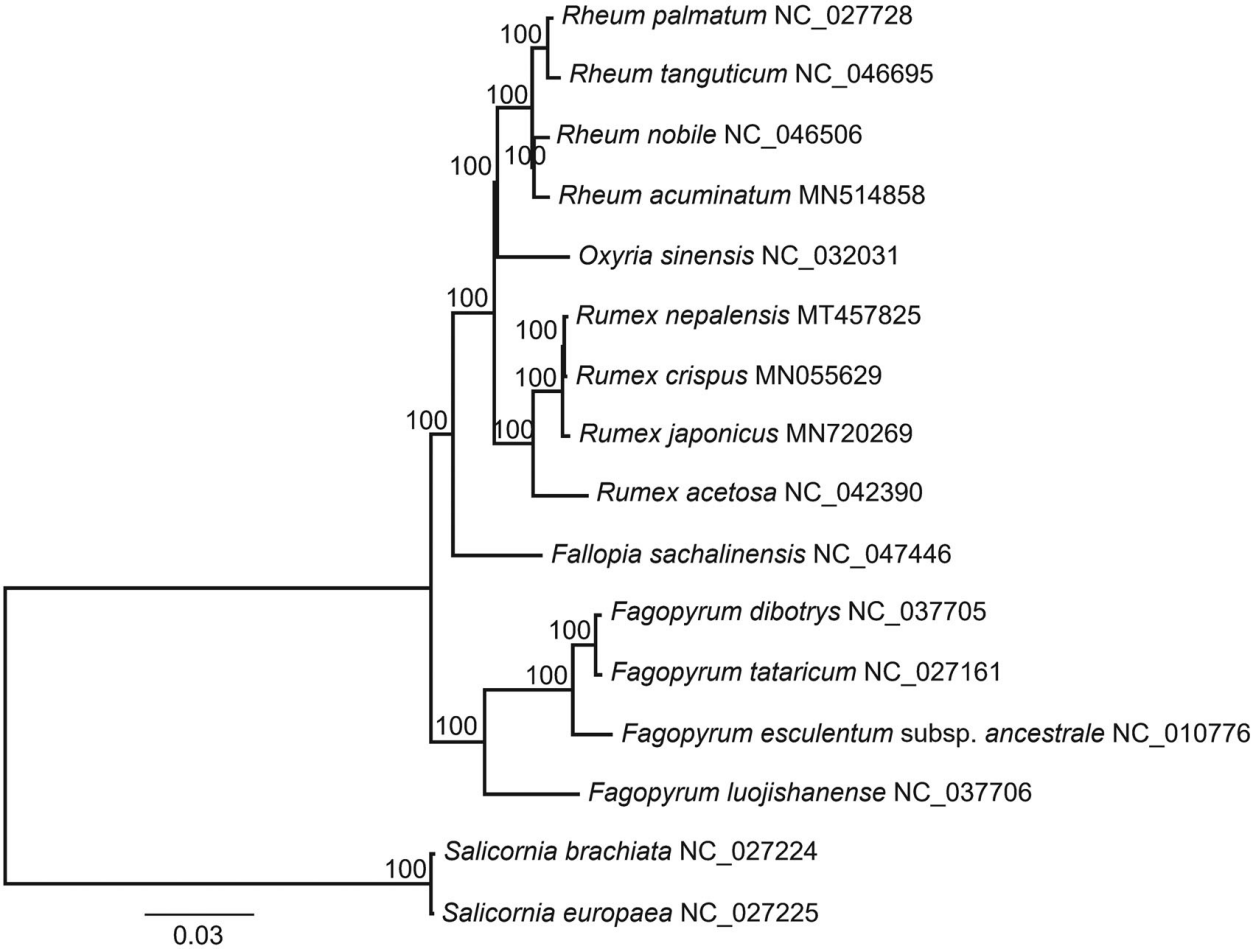
A maximum likelihood tree of 14 Polygonaceae species based on alignment of all plastome genes. *Salicornia brachiata* and *S. europaea* are used as outgroup. The numbers on branches are bootstrap support values.

## Data Availability

The data that support the findings of this study are openly available in GenBank of NCBI at https://www.ncbi.nlm.nih.gov, reference number MT457825.
